# The contribution of inflammasome components on macrophage response to surface nanotopography and chemistry

**DOI:** 10.1038/srep26207

**Published:** 2016-05-18

**Authors:** Susan Christo, Akash Bachhuka, Kerrilyn R. Diener, Krasimir Vasilev, John D. Hayball

**Affiliations:** 1 Experimental Therapeutics Laboratory, Sansom Institute and Hanson Institute, School of Pharmacy and Medical Science, University of South Australia, Adelaide, SA, 5000, Australia; 2 Mawson Institute, University of South Australia, SA, 5095, Australia; 3 Robinson Research Institute, School of Paediatrics and Reproductive Health, University of Adelaide, Adelaide, SA, 5005, Australia; 4 School of Engineering, University of South Australia, SA, 5095, Australia; 5 School of Medicine, University of Adelaide, Adelaide, SA, 5005, Australia

## Abstract

Implantable devices have become an established part of medical practice. However, often a negative inflammatory host response can impede the integration and functionality of the device. In this paper, we interrogate the role of surface nanotopography and chemistry on the potential molecular role of the inflammasome in controlling macrophage responses. To achieve this goal we engineered model substrata having precisely controlled nanotopography of predetermined height and tailored outermost surface chemistry. Bone marrow derived macrophages (BMDM) were harvested from genetically engineered mice deficient in the inflammasome components ASC, NLRP3 and AIM2. These cells were then cultured on these nanoengineered substrata and assessed for their capacity to attach and express pro-inflammatory cytokines. Our data provide evidence that the inflammasome components ASC, NLRP3 and AIM2 play a role in regulating macrophage adhesion and activation in response to surface nanotopography and chemistry. The findings of this paper are important for understanding the inflammatory consequences caused by biomaterials and pave the way to the rational design of future implantable devices having controlled and predictable inflammatory outcomes.

The use of biomedical implants for improving or restoring biological tissue functionality is a rapidly expanding area within the medical field. However, with an estimated 20 million surgical and non-surgical implant procedures conducted globally^1^, there are risks of implant rejection, which increases the health and economic burden to the patient and society. Although the causes of implant failure are varied, they may be attributed to poor host integration and localized reactions. It has been well documented that implantable biomaterials have the potential to induce the foreign body response (FBR); an acute inflammatory reaction that overlaps with tissue remodeling and results in the fibrotic encapsulation of the device^2^. Ongoing attempts at reducing the adverse events of the FBR include controlling the immune inflammatory response, as well as modulating the nanotopography of the biomaterial surface. Model surface nanotopographies such as well-ordered and organized pores, grooves, pillars and colloidal particles^3^,^4^,^5^,^6^ have been evaluated for their ability to modulate cell adhesion, proliferation and differentiation^3^,^7^,^8^. Innate immune effector cells that are involved in the FBR are of particular interest^7^,^9^,^10^,^11^, specifically the functionality of macrophages that are known to present multiple phenotypes based on cytokine production. In general, studies have demonstrated that surfaces with smaller pores can increase macrophage adhesion, cytoskeletal morphology^12^ and cytokine expression, whilst reducing reactive oxygen species production^13^,^14^. Similarly, microfabricated grooves and steps increased macrophage adhesion, elongation and secretion of pro-inflammatory cytokines when compared to planar surfaces^4^,^15^,^16^. However, alternative surface perturbations such as carbon and titanium nanotubes have shown to reduce macrophage adhesion and proliferation^9^,^17^,^18^,^19^,^20^.

Despite these important findings, the exact molecular mechanisms that underpin differential macrophage responses on surface nanotopographies remain to be elucidated. Recent studies have focused on the potential molecular control of the FBR by the inflammasome; a multi-protein complex that regulates the production of the potent cytokine, IL-1β^21^. The inflammasome is activated when sensor proteins are triggered by their stimuli, such as foreign pathogens or cell damage. This then recruits the ASC adaptor molecule, followed by pro-caspase-1 that together forms a permissive geometry for the proteolytic cleavage of pro-caspase-1 into caspase-1. Caspase-1 acts to cleave pro-IL-1β into its active IL-1β form^22^. Evidence suggests that the inflammasome may be activated upon biomaterial implantation, in part, as an extension to the knowledge that non-phagocytosable particles, such as asbestos and silica, activate the NLRP3 inflammasome specifically^23^. The NLRP3 inflammasome can also be triggered by nanoparticular carbon^24^ and polystyrene^25^, as well as nanodebris typically derived from implants^26^ including amorphous silica and titanium dioxide (TiO_2_)^27^, CoCrMo^28^, and silver^29^,^30^. However, efforts to assess the inflammasome response to synthetic materials do not take into account, nor controllably evaluate, the effect of surface topography on modulating innate immune cell functionality. Therefore, the purpose of this study was to investigate the potential role of inflammasome components in modulating macrophage functionality when exposed to surfaces of controlled nanotopography.

To achieve this goal, we generated model substrata having controlled nanotopography of predetermined height and tailored outermost surface chemistry. Macrophages were harvested from genetically engineered mice deficient in the inflammasome components ASC, NLRP3 and AIM2, and were then cultured on these substrata to assess cell adhesion and functionality. It was found that cells deficient in ASC and NLRP3, but not AIM2, had reduced adhesion on surface nanotopography and chemistry. When pro-inflammatory cytokine secretion was assessed as a surrogate of macrophage functionality, it was found that ASC^−/−^ and NLRP3^−/−^ BMDM had reduced IL-1β levels and higher TNF-α and IL-6, which was in contrast to patterns produced by AIM2^−/−^ BMDM. Therefore, whilst the mechanistic basis of these results remain to be determined, modulation of BMDM activity in the absence of ASC, NLRP3 and AIM2 suggest that these inflammasome components, in part, may play a role in macrophage responses to surface nanotopography and chemistry.

## Results

### The generation of nanotopographically modified surfaces

**Figure 1:**
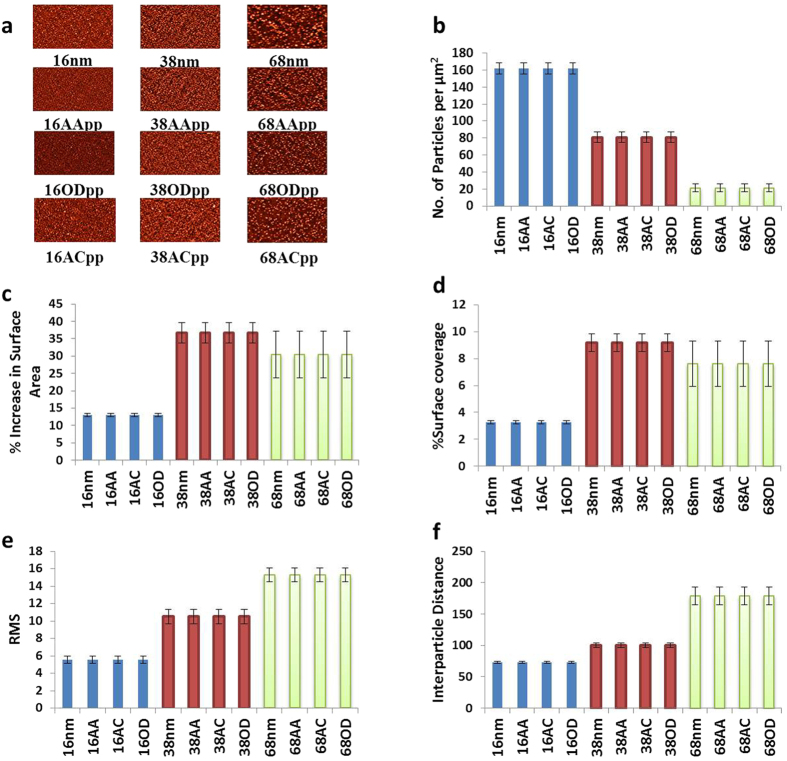
Atomic force microscopic analysis of surfaces modified with 16, 38 or 68 nm gold nanoparticles. (**a**) Two dimensional AFM images of gold nanoparticles overcoated with a 5 nm thin layer of allylamine (AA). (**b**) The number of particles per μm^2^, (**c**) percentage increase in surface area, (**d**) percentage surface coverage, (**e**) root mean squared (RMS), and (**f)** interparticle distance were calculated for surface modified with 16, 38 and 68 nm gold nanoparticles.

Surfaces of controlled nanotopography were generated by first modifying a 13 mm glass coverslip with a 20 nm thin layer of plasma deposited allylamine (AApp). These type of coatings are known to be rich in amine functional groups which carry a positive surface charge in aqueous medium below pH = 8^ ^31,32. To generate surface nanotopography, gold nanoparticles (AuNPs) carrying carboxyl acid functionalities and having diameters of 16 nm, 38 nm, and 68 nm were electrostatically immobilized on the amine modified coverslips. The role of the gold nanoparticles is to provide surface nanotopography of controlled z-scale. Finally, to provide a tailored surface chemistry, a 5 nm thin layer deposited from plasma of AApp, octadiene (ODpp) or acrylic acid (ACpp) was applied on top of the nanoparticles modified surfaces. The selection of plasma polymer film thickness was based on our earlier finding which demonstrate that 5 nm provides continuous and uniform coatings but allows us to preserve the scale of surface nanotopography^33^,^34^. Together, this process resulted in three different scales of surface nanotopography and known and tailored outermost surface chemistry. Images of the generated model substrata before and after overcoating are shown in Fig. 1a.

Characterization of the surface nanotopography in terms of nanoparticles per micrometer square, surface area, RMS and interparticle distance was carried out from the AFM images and is shown in Fig. 1b–f. The number of particles varied from 162 to 21 per μm^2^ for 16 nm to 68 nm AuNPs, respectively (Fig. 1b). When the percentage increase in surface area was calculated (Fig. 1c), it was found that it increased from 13% to 36% for 16 nm and 38 nm AuNPs, respectively; however there was a drop to 30% for 68 nm AuNPs (Fig. 1c). The percentage increase in surface coverage of AuNPs was also calculated and found to increase from 3% to 8% for 16 nm and 38 nm AuNPs, respectively, and decreased slightly to 7.5% for 68 nm AuNPs (Fig. 1d). The variation of the percentage surface area and surface coverage increases can be attributed to the differences in total number of particles attached to the surfaces. The root mean square roughness (RMS) revealed a directly proportional increase with AuNPs size such that RMS increases from 5 nm to 15 nm on 16 nm and 68 nm AuNPs, respectively (Fig. 1e). The interparticle distance also increased on surfaces with larger AuNPs, with a distance of 72 nm and 179 nm between 16 nm and 68 nm AuNPs, respectively (Fig. 1f).

**Figure 2:**
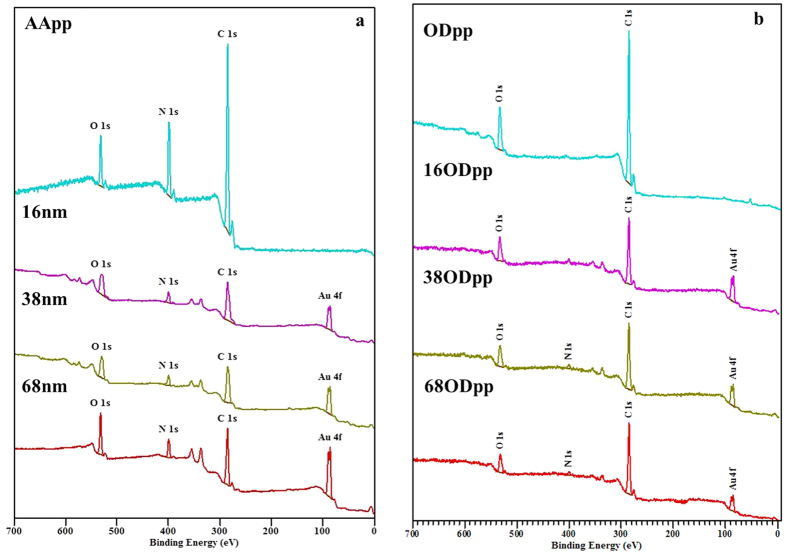
XPS analysis of controlled nanotopography surfaces. (**a**) Survey spectra of allylamine coated glass coverslip and glass coverslips modified with 16 nm, 38 nm and 68 nm nanoparticles. (**b**) Survey spectra of octadiene (OD) coated glass coverslip and nanoparticles overcoated with octadiene 16OD, 38OD and 68OD.

The surface chemical composition of all model substrata was analyzed by XPS (Fig. 2). Figure 2a shows survey spectra of a glass coverslip modified with a layer of AApp only and after immobilization of gold nanoparticles. The AApp coatings shows three distinct peaks corresponding to carbon, oxygen and nitrogen and is consistent with published data^34^. The absence of signal from the substrate (i.e. silicon) suggests that the coating is continuous and pinhole-free, and the thickness is greater than the sampling depth of XPS (ca 10 nm). Upon AuNPs immobilization, a district peak corresponding to Au 4f is detectable. Other notable changes in the spectra are an increase in O/C ratio which could be traced to the COOH surface functionality of the gold nanoparticles. Figure 2b show survey spectra of the substrates with immobilized gold nanoparticles after overcoating with 5 nm thin layer of ODpp and the survey spectrum of the ODpp control. The ODpp coating consist of carbon only, however some oxidation upon exposure to ambient atmosphere also occur. When the immobilized gold nanoparticles are overcoated with ODpp, a signal corresponding to Au 4f is still detectable because the film is thinner than the sampling depth of XPS. For the same N1s signal originating from the underlying AApp coating is also detectable. Similar trends were observed for AApp and ACpp overcoatings and are shown in Supplementary Figure 1. The surface chemical composition of all samples which is summarized in Supplementary Table 1. Taken together, 12 substrates of controlled and characterized nanotopography and tailored outermost surface chemistries were generated for assessing their potential impact on inflammasome-dependent macrophage functionality.

### Assessing the effect of controlled surface nanotopography and inflammasome components on macrophage-derived cytokine secretion

**Figure 3:**
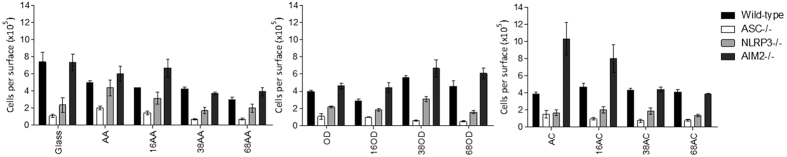
Bone marrow-derived macrophage (BMDM) adhesion on controlled nanotopography surfaces. BMDM cultured from wild-type, ASC^−/−^, NLRP3^−/−^ or AIM2^−/−^ mice were incubated on controlled nanotopography surfaces before being fixed, stained with DAPI for the quantification of nuclei to deduce the total cell numbers per surface. Results are expressed as mean ± SEM and are representative of two independent experiments. The p values were calculated in reference to the glass control surfaces for each murine genotype using a one way analysis of variance with a Dunnett’s post-test. There was no significance obtained amongst the comparisons (p < 0.05).

To determine the potential role of the combination of surface chemistry and nanotopography on macrophage responses, the number of bone-marrow derived macrophages (BMDM) on the surfaces was quantified for all murine genotypes. It was found that the numbers of adherent wild-type BMDM incubated on controlled nanotopography surfaces was reduced regardless of surface chemistry or nanotopography scale compared to a bare glass. To determine if there was a role of the inflammasome in modulating the ability of macrophages to remain on controlled nanotopography surfaces, numbers of wild-type BMDM and BMDM deficient in the common inflammasome mediator ASC, were quantified (Fig. 3). It was found that a dramatic decrease in ASC^−/−^ BMDM adhesion incubated on nanotopography modified surfaces compared to planer controls having the same chemistry or glass control surfaces. A similar reduction was observed for BMDM deficient in the NLRP3 sensor protein in the case of amine modified surfaces; however, when AC and OD chemistries were used, there were no significant trends in terms of number of adherent cells, with the exception of an increased cell adhesion on AA surfaces compared to the glass control surface (Fig. 3). The role of an additional inflammasome sensor, AIM2 was also assessed because we recently observed its potential contribution to collagen production during an *in vivo* model of the FBR^35^. The AIM2 inflammasome is triggered by extracellular DNA from foreign invaders, but may also detect self-DNA released from apoptotic cells during cell damage. It was observed that the adhesion of AIM2^−/−^ BMDM were reduced in a similar pattern to that of wild-type BMDM, however AC and 16AC surfaces did not appear to affect AIM2^−/−^ BMDM adhesion as their numbers were comparable to that observed on glass control (Fig. 3).

**Figure 4:**
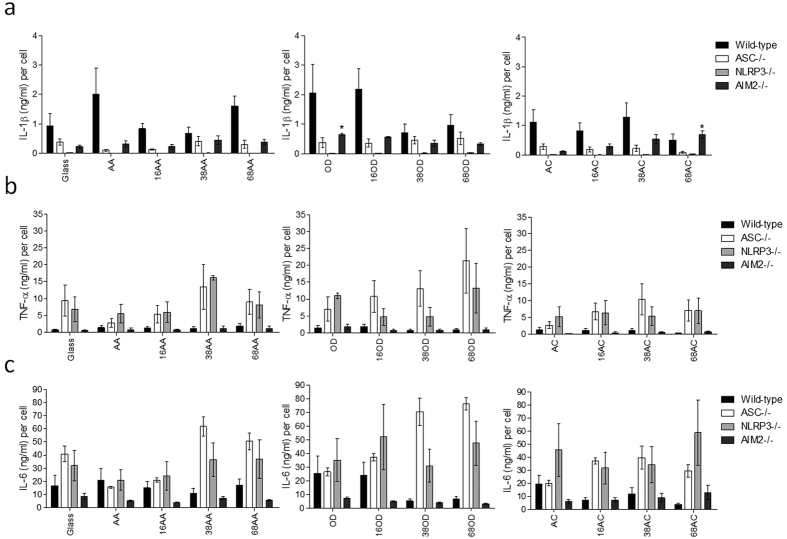
Secretion of pro-inflammatory cytokines from BMDM incubated on controlled nanotopography surfaces. BMDM cultured from wild-type, ASC^−/−^, NLRP3^−/−^ or AIM2^−/−^ mice were incubated on controlled nanotopography surfaces before being activated with LPS and ATP for the detection of IL-1β (**a**) or activated with LPS for the detection of TNFα (**b**) and IL-6 (**c**). Cytokine levels were expressed as cytokine (ng/ml) per cell to account for differences in the numbers of adherent cells. Results are expressed as mean ± SEM and are representative of three independent experiments. The p values were calculated in reference to the glass control surfaces for each murine genotype using a one way analysis of variance with a Dunnett’s post-test. *p < 0.05.

To then determine the functionality of macrophages incubated on controlled nanotopography surfaces, the production of pro-inflammatory cytokines implicated in the FBR was measured (Fig. 4). Secretion of IL-1β (Fig. 4a), TNFα (Fig. 4b) and IL-6 (Fig. 4c) were expressed as cytokine concentration secreted per cell to account for the differences in the number of adherent BMDM. Despite being not statistically significant, IL-1β secretion by individual cells was doubled when wild-type BMDM were incubated on AA, OD and 16OD surfaces when compared to glass control (Fig. 4a). However, levels were decreased by 50% when incubated on the hydrophilic anionic 68AC surfaces. In the case of ASC^−/−^ BMDM, there were diminished levels of IL-1β regardless of surface type, and this was attributed to NLRP3 stimulation as observed by the negligible IL-1β secretion in NLRP3^−/−^ BMDM. Interestingly, although AIM2^−/−^ BMDM secreted 75% less IL-1β on a per cell basis compared to wild-type BMDM on glass surfaces, there was a statistically significant three-fold increase in IL-1β on OD and 68AC surfaces. A similar three-fold increase was observed on 16OD and 38AC surfaces.

To then assess if other pro-inflammatory cytokines were modulated by controlled nanotopography, TNFα (Fig. 4b) and IL-6 (Fig. 4c) were analyzed in activated BMDM incubated on the surfaces. Interestingly there was an observable increase in TNFα secretion per cell by ASC^−/−^ and NLRP3^−/−^ BMDM incubated on all surfaces compared to wild-type BMDM, and in the case of ASC^−/−^ BMDM, TNFα levels were maximal on 68OD surfaces, but reduced on AA and AC surfaces. Similar patterns were seen for IL-6 secretion per cell, with ASC^−/−^ BMDM producing higher levels on glass surfaces when compared to wild-type BMDM, which were enhanced on 38AA, 38OD and 68OD and reduced on AA and 16AA surfaces. NLRP3^−/−^ BMDM were mostly secreting greater levels of IL-6 per cell compared to wild-type BMDM, whereas AIM2^−/−^ BMDM were producing less IL-6 across the various surfaces.

## Discussion

**Figure 5:**
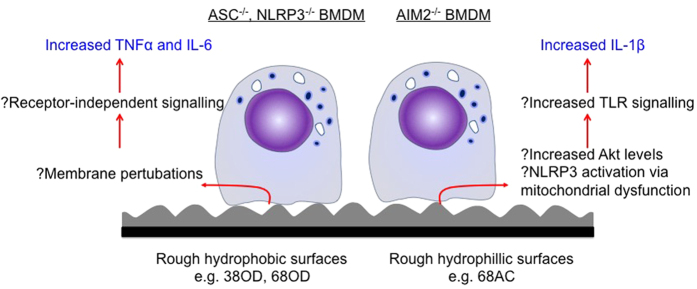
Schematic representation of the hypotheses proposed for the involvement of inflammasome components ASC, NLRP3 and AIM2 in macrophages responses to controlled nanotopography and chemistry. We hypothesise that the increased levels of TNFα and IL-6 by ASC^−/−^ and NLRP3^−/−^ BMDM is due to membrane perturbations caused by the curvature of the nanoparticles, leading to Syk kinase activation as proposed by the membrane affinity triggered signalling model. This receptor-independent signalling may result in higher cytokine levels in the absence of inflammasome components capable of IL-1β secretion. In the case of AIM2^−/−^ BMDM, we hypothesise that cell perturbations on ‘rougher’ surfaces result in NLRP3 activation via mitochondrial dystunction, and in addition to potentially higher levels of Akt in the absence of AIM2, these pathways enhance TLR signalling that control IL-1β release. The black text represents our hypotheses and the blue text represents the observations of this study.

In this study, surfaces modified with well-defined nanotopographies and tailored uniform outermost chemistries were utilized to determine the molecular role of the inflammasome in the interaction between macrophages and synthetic surfaces. By combining electrostatic immobilization of gold nanoparticles to generate controlled height of surface nanotopography, followed by overcoating with various but uniform chemistries, a set of 12 different model substrata were developed. These surfaces were then assessed for their effects on macrophages, which prominent innate immune effector cells involved in the FBR. Macrophage adhesion was altered on all controlled nanotopography surfaces regardless of surface chemistry or nanotopography scale when compared to glass control. Despite the understanding that various surface chemistries can affect the initial binding of serum proteins that then influence cell attachment^8^,^36^,^37^,^38^,^39^, there did not appear to be obvious differences amongst the surface types. However, there did appear to be changes to macrophage functionality suggesting that the cells were able to sense and respond to the various surface phenotypes particularly in the absence of key inflammasome components. The striking increases in TNFα production by ASC^−/−^ and NLPR3^−/−^ BMDM may be due to compensatory mechanisms that are activated in the absence of IL-1β secretion^40^ and mostly likely occur in an NFκB-dependent manner upon LPS stimulation. However, the enhanced secretion of TNFα and IL-6 by ASC^−/−^ BMDM on 38OD and 68OD surfaces above that of glass control suggests that these surfaces were able to stimulate additional pathways that led to cytokine expression. To this end, recent reports have proposed the membrane affinity triggered signaling mode of activation. This model suggests that disturbances to membrane curvature can lead to membrane lipid re-arrangement to activate Syk kinase-dependent signaling^41^,^42^. Therefore, based on the observations in this study it can be hypothesized that the combination of hydrophobic OD chemistry and surface nanotopography alters the membrane conformation of ASC^−/−^ BMDM to potentially activate receptor-independent signaling. However, this does not take into account differences in surface chemistry as similar results were not observed on hydrophilic 38AC or 68AC surfaces. The schematic in Fig. 5 highlights our proposed hypothesis (black text) based on the given observations (blue text).

It has been previously demonstrated that hydrophilic surfaces do not bind serum proteins as strongly as hydrophobic surfaces and that these proteins will desorb faster^43^, which may affect cell binding. In contrast to this, the increased adhesion of AIM2^−/−^ BMDM on AC and 16AC surfaces did not appear to affect their functionality. However, it was surprising that IL-1β secretion was reduced on all surface types, suggesting that the AIM2 inflammasome does play a role in responding to controlled nanotopography surfaces. Interestingly, 68AC surfaces had the highest levels of IL-1β production per cell despite the lowest number of adherent AIM2^−/−^ BMDM. This may suggest that 68AC surfaces induced cell damage and apoptosis, which led to NLRP3 activation via mitochondrial dysfunction^44^. In support of this hypothesis, studies have demonstrated reduced macrophage adhesion and increased apoptosis on hydrophilic anionic surfaces^45^,^46^. Furthermore, an inflammasome-independent role for AIM2 was recently detailed in the context of colon cancer, suggesting that AIM2 can inhibit the Akt kinase involved in proliferative pathways^47^,^48^. It is therefore tempting to speculate that BMDM have increased levels of Akt, which is downstream to TLR signaling^49^, thereby enhancing ‘signal 1’ and generating higher levels of IL-1β (Fig. 5). Whilst the mechanistic basis of these results remain to be determined, this study has shown previously undescribed observations that inflammasome components, ASC, NLRP3 and AIM2 do play a role in macrophage responses to surface nanotopography and chemistry.

This work addresses the role of surface nanotopography and chemistry on the potential molecular role of the inflammasome in controlling macrophage responses. Models substrata having controlled nanotopography and chemistry were generated by combining plasma polymerisation and electrostatic self-assembly of gold nanoparticles controlled and predetermined diameters. BMDM were harvested from genetically engineered mice deficient in the inflammasome components ASC, NLRP3 and AIM2, which were then cultured on these substrata and assessed for their capacity to attach and express pro-inflammatory cytokines. The data demonstrates that inflammasome components ASC, NLRP3 and AIM2 may play a role in regulating macrophage adhesion and activation in response to the combination of surface nanotopography and chemistry. The findings of this paper are important to understanding the inflammatory consequences caused by biomaterials. It may also pave the way towards the rational design of advanced implantable devices having controlled and predictable inflammatory outcomes.

## Methods

### Materials

Allylamine (AA) (98%, Aldrich), acrylic acid (AAC) (99%, Aldrich), octadiene (OD) (98%, Aldrich), hydrogen tetrachloroaurate (99.9985%, ProSciTech), trisodium citrate (99%, BHD Chemicals, Australia Pty. Ltd.), 2-mercaptosuccinic acid (97%, Aldrich), Poly (Styrene Sulphonate) (Aldrich) were used as received.

### Plasma polymerization

Plasma polymerization was carried out in a custom built reactor with a 13.56 MHz plasma generator^23^. Deposition of allylamine, acrylic acid and octadiene was carried out at a pressure of 0.2 mbar and a deposition time of 2 min was employed. Power used for deposition of all three monomers was 40 W, 10 W and 20 W respectively. Using these conditions we obtained polymer film of thickness of 23 nm, 20 nm and 25 nm for films deposited from allylamine, acrylic acid and octadiene, respectively. Before deposition, all substrates were cleaned by applying an air plasma for 2 min at 50 W.

### Synthesis of gold nanoparticles (AuNPs)

Gold (Au) nanoparticles (NPs) were synthesized by reducing hydrogen tetrachloroaurate (HAuCl_4_) using trisodium citrate, before a 0.01% HAuCl_4_ solution was boiled with vigorous stirring. Particles of 16, 38 and 68 nm diameter were synthesized by varying the amount of 1% trisodium citrate from 1 ml to 0.3 mL, respectively^50^, which altered the color of the solutions from light yellow to wine red. The solution was boiled for an additional 20 min before being left to cool down to room temperature^50^, and then surface modification of these nanoparticles was performed by using 2-mercaptosuccinic acid as described elsewhere^51^.

### Immobilization of gold nanoparticles (AuNPs)

For immobilization, plasma polymerised allylamine (AA) samples were immersed in a solution of gold nanoparticles for different time intervals ranging from 2 to 15 hrs depending on the size of particles. These surfaces when immersed in solution carry positive charge while nanoparticles are capped with negatively charged carboxylic acid. This lead to an electrostatic binding between positively charged AA and negatively charged gold nanoparticles. For our experiments, we immersed AA in 3 different sized gold nanoparticles (16, 38 and 68 nm). Finally, to remove loosely bound nanoparticles these samples were washed with Milli-Q water and dried using nitrogen.

### Atomic Force Microscopy (AFM)

An NT-MDT NTEGRA SPM atomic force microscope (AFM) was used in non-contact mode to provide nanotopographical images. Au coated silicon nitride tips were used in non-contact mode on the reflective side (NT-MDT, NSG03) and had resonance frequencies between 65 and 100 kHz. Images of 5 μm × 5 μm were scanned at a scan rate of 0.5 Hz and amplitude of oscillation of 10nm. Image J was employed for calculating number of particles from these AFM images. The number of particles was used to calculate percentage increase in surface area, percentage surface coverage and interparticle distance. For calculating number of nanoparticles per μm^2^, % increase in surface area, % surface coverage, and interparticle distance we have prepared three samples per nanoparticle size. These samples were analyzed by taking three images per sample.

### X-ray Photoelectron Spectroscopy

XPS was used to determine the surface composition of the plasma polymers with the deposited AuNPs. Spec SAGE XPS spectrometer equipped with a monochromatic Mg radiation source was operated at 10 kV and 20 mA to record all spectra. Survey spectra were recorded at pass energy of 100 eV and 0.5 eV resolution for identifying the atomic concentrations of all samples. All binding energies (BE) were corrected relative to a neutral C1s carbon peak at 285.0 eV. Processing and curve fitting was performed in Casa XPS.

### Animals

All experimental protocols were approved by the Animal Ethics Committees of SA Pathology and the University of Adelaide, and all methods were performed in accordance to the guidelines of the University of South Australia (South Australian Animal Welfare Act 1985). Wild-type C57Bl/6 (B6) mice were purchased from Laboratory Animal Services (University of Adelaide, SA, Australia). Mice deficient in ASC were kindly provided by Professor Vishwa Dixit, Yale University. Mice deficient in NLRP3 were kindly provided by Professor Hal Hoffman, University of California, San Diego. Mice deficient in AIM2 were kindly provided in Professor Kate Fitzgerald, University of Massachusetts. Animals were housed in the Reid Animal Facility (University of South Australia) under pathogen-free conditions.

### Preparing L929 conditioning media

To prepare L929 conditioned media, confluent L929 cells were detached, collected and passaged 1:10. Cells were then cultured until the media was exhausted (7–8 days). The conditioned media, which contained the macrophage growth factor M-CSF, was removed, filtered (0.22 μm), aliquoted and stored at −20 °C until required.

### *In vitro* culture of bone marrow derived macrophages

Bone marrow derived macrophage (BMDM) cells were generated by flushing bone marrow cells from femurs and tibia of B6 mice, depleting red blood cells using lysis buffer, and resuspending cell in complete RPMI supplemented with 20% L929 conditioned media. On day 4 of culture, suspension cells were removed, and media replenished before being seeded on coverslips modified with the indicated surface nanotopography. Cells were maintained for an additional three days at 37 °C/5%C02. To assess BMDM phenotype, cells were gently scraped off on day 7 of culture and stained with anti-CD11b and anti-F4-80 antibodies for 30 min on ice. Cells were washed, fixed and later ran on the flow cytometer for detection of CD11b+ F4–80+ events using the FlowJo software. This method allowed bound monocytes to differentiate into macrophages on the various surfaces for three days before external stimuli were added to assess how surface interactions influenced known macrophage function.

### Cytokine secretion from activated macrophages

On day 7, the *in vitro* generated BMDMs were stimulated with lipopolysaccharide (LPS, serotype O111:B4; 100 ng/mL; Sigma Aldrich) or left unstimulated for 4 hrs, and supernatant collected for the analysis of TNF-α, and IL-6. BMDMs were then stimulated with adenosine triphosphate (ATP, 5 mM; Sigma Aldrich) for 1 hr, and supernatant collected for IL-1β analysis using standard ELISA protocols. Cytokines were not detected in unstimulated cultures (data not shown).

### Quantifying numbers of adherent bone marrow derived macrophages

Day 7 BMDM plated onto surfaces were gently washed with PBS before fixing with overnight. Cells were stained with 1 μg/mL DAPI for 2 min at room temperature, and washed twice before mounting onto glass slide. Four images per surface were taken using an Olympus IX51 Fluorescence Microscope and the CellSens program. Image J software was used to automatically count the cells in the field of view, and the image dimensions (in μm) were used to calculate total cell numbers on the total area of the 13 mm-diameter glass coverslip.

### Statistical Analysis

All statistical analysis was performed on GraphPad Prism 5, and a one-way analysis of variance (ANOVA) performed with a Dunnett’s posttest. Each surface nanotopography was compared against the glass surfaces, which was selected as the control.

## Additional Information

**How to cite this article**: Christo, S. *et al*. The contribution of inflammasome components on macrophage response to surface nanotopography and chemistry. *Sci. Rep*. **6**, 26207; doi: 10.1038/srep26207 (2016).

## Supplementary Material

Supplementary Information
