# Supplementary material for: The contribution of inflammasome components on macrophage response to surface nanotopography and chemistry
Source: Sci Rep. 2016 May 18;6:26207. doi: 10.1038/srep26207 (PMC4870632; doi:10.1038/srep26207)
Supplement: Supplementary Information [file srep26207-s1.pdf]

SUPPLEMENTARY INFORMATION

The contribution of inflammasome components on macrophage response to surface nanotopography and chemistry

Susan N. Christo[a, #] Akash Bachhuka[b, #] Kerrilyn R. Diener[c], Krasimir Vasilev[a,d,*] and John D. Hayball[b,e,*.]

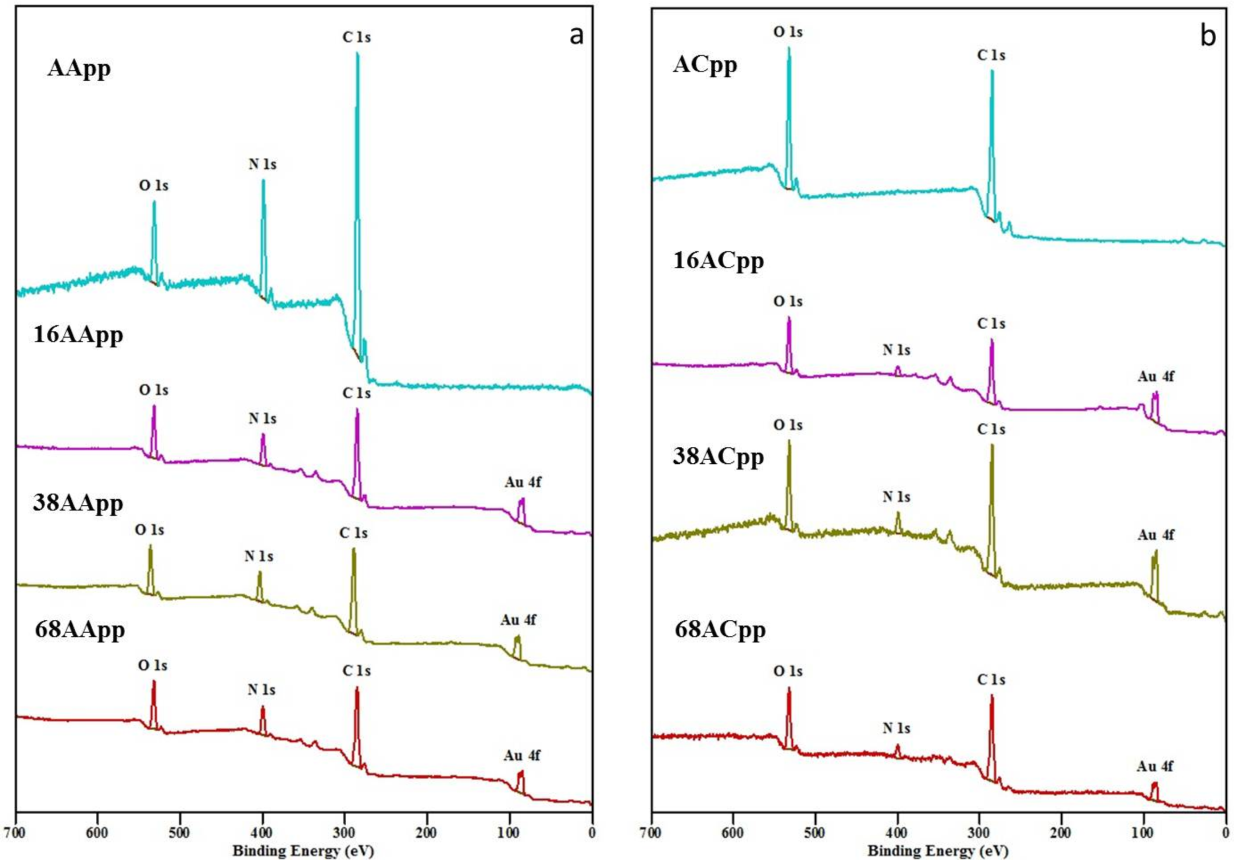

Supplementary Figure S1. XPS analysis of controlled nanotopography surfaces. (a) Survey spectra showing allylamine coated glass coverslips (AA) and nanoparticles overcoated with allylamine 16AA, 38AA and 68AA. (b) Survey spectra of acrylic acid coated glass coverslip (AC) and nanoparticles overcoated with acrylic acid16AC, 38AC and 68AC.

Supplementary Table S1. Surface chemical composition of different nanotopography modified surface derived from the XPS analysis. All the XPS data was plotted with a standard error of 5%.

Surface	C1s	N1s	O1s	Si2p	Au (At %)	Au/C	N/C	O/C
**GCS**	19	-	40	41	-	-	-	2.1
**16 nm**	71.9	8.2	17.6	-	2.3	0.03	0.11	0.25
**38 nm**	71.8	8.5	16.7	-	3.1	0.04	0.12	0.2
**68 nm**	67.6	10.8	17.1	-	4.3	0.06	0.16	0.18
**AApp**	75.8	16.2	8.0	-	-	-	0.21	0.11
**16AApp**	71.1	13.0	14.4	-	1.5	0.02	0.18	0.18
**38AApp**	71.5	12.9	14.2	-	1.4	0.02	0.18	0.19
**68AApp**	71.5	13.0	14.1	-	1.4	0.02	0.18	0.18
**ODpp**	88.1	-	11.9	-	-	-	-	0.14
**16ODpp**	83.2	-	14.3	-	1.8	0.02	-	0.15
**38ODpp**	82.5	2.5	13.1	-	1.9	0.02	0.03	0.16
**68ODpp**	85.5	2.4	10.7	-	1.4	0.02	0.02	0.13
**ACpp**	76.6	-	23.4	-	-	-	-	0.31
**16ACpp**	70.8	6.1	20.6	-	1.5	0.02	0.09	0.29
**38ACpp**	72.4	3.4	23.3	-	0.9	0.01	0.05	0.29
**68ACpp**	74.0	6.0	18.8	-	1.2	0.02	0.08	0.29
